# The Use of Pigs as a Translational Model for Studying Neurodegenerative Diseases

**DOI:** 10.3389/fphys.2019.00838

**Published:** 2019-07-10

**Authors:** Brendan Hoffe, Matthew R. Holahan

**Affiliations:** Department of Neuroscience, Carleton University, Ottawa, ON, Canada

**Keywords:** neurodegenerative diseases, Alzheimer’s disease, traumatic brain injury, porcine model, translational science, large animal models

## Abstract

In recent years, the move to study neurodegenerative disease using larger animal models with brains that are more similar to humans has gained interest. While pigs have been used for various biomedical applications and research, it has only been recently that they have been used to study neurodegenerative diseases due to their neuroanatomically similar gyrencephalic brains and similar neurophysiological processes as seen in humans. This review focuses on the use of pigs in the study of Alzheimer’s disease (AD) and traumatic brain injury (TBI). AD is considered the most common neurodegenerative disease in elderly populations. Head impacts from falls are the most common form of injury in the elderly and recent literature has shown an association between repetitive head impacts and the development of AD. This review summarizes research into the pathological mechanisms underlying AD and TBI as well as the advantages and disadvantages of using pigs in the neuroscientific study of these disease processes. With the lack of successful therapeutics for neurodegenerative diseases, and an increasing elderly population, the use of pigs may provide a better translational model for understanding and treating these diseases.

## Introduction

In 2010, 35 million individuals worldwide suffered from dementia, with this number to only increase in developed countries as the baby boomer generation begins to reach the senior years of life (Wimo et al., 2013). The cost of caring for those suffering from dementia was estimated to be over $200 billion USD in North America (Wimo et al., 2013). Alzheimer’s disease (AD) is considered the most prominent neurodegenerative disorder as it accounts for nearly 80% of dementia cases world-wide (Kumar et al., 2015). The availability of appropriate pharmacotherapeutics to treat AD pathology has not kept pace with this rise in prevalence. In 2014, a meta-analysis of clinical trials for AD therapeutics found the success rate of stage 3 approval was 0.4% (Cummings et al., 2014).

Gaining a more complete understanding of the etiology of AD may provide insight to guide the development of target-specific AD treatments. While there are a number of potential contributing factors that culminate in AD pathology, epidemiological studies indicate traumatic brain injury (TBI) as an important predisposing factor (Van Den Heuvel et al., 2007). In 2017, it was reported that TBIs were among the leading causes of injury in the elderly (Taylor et al., 2017). In recent years, TBI has been shown to be associated with the development of an Alzheimer’s-like pathology known as chronic traumatic encephalopathy (CTE; McKee et al., 2013). Therefore, a fundamental understanding of the link between TBI and neuropathology may be instrumental in guiding AD treatment development.

Historically, rodents have been the primary model system in the exploration of the mechanisms behind AD and TBI. Rodents are inexpensive, well researched, and can be genetically modified to produce a wide variety of genotypic backgrounds. However, with the extremely low clinical trial success rate, there is a need for better translational science to aid in the development of therapeutics for the vulnerable aging population (Jakobsen et al., 2016). In the past decade, there has been increased interest in using large animals to better understand the mechanisms behind neurodegenerative diseases such as AD and TBI (Lind et al., 2007; Roth and Tuggle, 2015). One large animal of particular interest is pigs (for review of using pigs in different neurodegenerative diseases, see Dolezalova et al., 2014). This review will focus specifically on the similarities and connection between AD and TBI pathology and why a pig model of these diseases could benefit the development of therapeutics.

## Alzheimer’s Disease Pathology

### β-Amyloid

AD is characterized by the progressive deterioration of the brain resulting in a decline in cognitive and behavioral abilities (McKhann et al., 1984; Scheff et al., 2006). Two key neuropathological characteristics include the build-up of β-amyloid plaque deposits (Aβ) and the aggregation of fibrous material within the neurons known as neurofibrillary tangles (NFT; Braak and Braak, 1991; Sabri et al., 2015). Aβ is the product of the transmembrane protein amyloid precursor protein being improperly cleaved by β and γ secretases (Wilson et al., 1999; Tu et al., 2014). This improper cleavage results in the aggregation of Aβ_42_ and ultimately Aβ oligomers (Tanzi, 2012; Okuda et al., 2017; Shu et al., 2018). The accumulation of Aβ has been shown to alter the firing of neurons (Palop and Mucke, 2010), increase intracellular Ca^2+^, reactive oxygen species production, and eventually apoptosis (Alberdi et al., 2010). In humans, this accumulation begins in the neocortex and gradually begins to affect areas of the hippocampus, followed by sensory and motor association cortices (Braak and Braak, 1991).

### Tau

While AD is commonly referred to as a multifactorial disease, the pathological progression has been hypothesized to start with the hyperphosphorylation of tau (Maccioni et al., 2010). Tau is a member of the microtubule associated protein (MAP) family responsible for the stability and structural integrity of microtubules (Maccioni and Cambiazo, 1995; Lucke-Wold et al., 2014). Mutations to the tau-encoding gene have been shown to lead to an increased likelihood of hyperphosphorylation and the development of NFTs (Iqbal et al., 2013; Miyashita et al., 2014). While the initial stages of hyperphosphorylation result in inhibition of microtubule function, the continuous phosphorylation of tau leads to accumulation and begins the development of NFTs (Iqbal et al., 2013). Phosphatases responsible for the dephosphorylation of tau cannot effectively clear the oligomers leading to the further increase in NFTs (Lucke-Wold et al., 2014). NFTs have been shown to lead to reactive oxygen species production and caspase signaling eventually leading to cell death (Arnaud et al., 2009; Sepulveda-Diaz et al., 2015). Interestingly, De Calignon et al. (2010) found caspase signaling occurs before NFT development. The authors speculated that caspase is responsible for cleaving tau, and this cleaved tau interacts with normal tau to further develop NFTs seen in AD.

## Traumatic Brain Injury as a Contributing Factor to Alzheimer’s Disease

TBI has been associated with the development of an Alzheimer’s-like pathology known as CTE (McKee et al., 2013). Commonly seen in athletes who participate in physical contact sports, such as hockey and football (Omalu et al., 2005; McKee et al., 2013; Kiernan et al., 2015), CTE has also been reported in military veterans (Goldstein et al., 2012) and patients with self-injurious behaviors such as head-banging (Geddes et al., 1999; Lee et al., 2017a). While there appears to be distinct differences between AD and CTE clinical appearance (McKee et al., 2013), the pathology between the two diseases is quite similar.

## Axonal Damage Following Traumatic Brain Injury

In the context of a TBI, the brain undergoes a rapid acceleration and deceleration in either a linear (front to back) or rotational (rotation of the neck and head) fashion following inertial forces suddenly placed on to the body or skull. Axonal and dendritic connections are subsequently stretched due to the mechanical forces within the brain, leading to swelling (Gennarelli et al., 1982). This swelling can be seen as early as 3 h after mechanical stretch *in vitro* (Tang-Schomer et al., 2012). Swelling of the axon and dendrite is often associated with transport and signal disruption as the microtubules begin to breakdown (Smith et al., 1999b). Stretching results in an increase in intracellular Ca^2+^ (von Reyn et al., 2009; Tao et al., 2017) that can lead to the activation of numerous biochemical pathways resulting in mitochondrial dysfunction, oxidative stress, reactive oxygen species production, and ultimately apoptosis (Kim et al., 2012). Along with activating apoptotic signals, increased Ca^2+^ activates calpain, an enzyme that has been shown to further activate proinflammatory processes (Tao et al., 2017) Under normal conditions, calpain is involved in numerous intracellular processes such as cytoskeleton remodeling, vesicular trafficking, signal transduction and apoptosis (for review, see Ma, 2013). The increase in Ca^2+^ from TBI mediates the cleavage of p35 to p25 which dimerizes with cdk5 (Yousuf et al., 2016). The cdk5/p25 complex phosphorylates microtubules within the cell, leading to neurotoxic effects and eventually apoptosis (Yousuf et al., 2016). Kim et al. (2016) demonstrated that the breakdown products of calpain promote the transformation of resting astrocytes into reactive astrocytes. These reactive astrocytes begin to clear debris of damaged neurons sustained from axonal injury; however, the exact mechanism of how this takes place still remains unclear (Blennow et al., 2016).

## Use of Porcine Models in the Study of Neural Function

Pigs have been utilized in a wide array of biomedical and biological research ranging from toxicology, to experimental surgery, to behavioral research (Richer et al., 1998; Swindle et al., 2012; Danek et al., 2017). Bustad and McClellan (1965) were one of the first to review the topic following the first symposium of pig use in biomedical research. Extensive research had already been underway before this symposium, including the demonstration a high degree of homology between pigs and humans. One major finding was the similarity of pig cardiac sections to those in humans; a finding still used today for valve transplantation (Manji et al., 2014). Other examples include: heat production and loss in piglets is similar to that of newborn humans; formation of spontaneous atherosclerosis lesions in pigs is comparable to humans in the pre-atheromatous phase of atherosclerosis; and, severe protein malnutrition in pigs can lead to biochemical and anatomical changes similar to what is seen in children suffering from kwashiorkor (Bustad and McClellan, 1965). Roth and Tuggle (2015) note that pigs are a better translational model for researching skin wound healing, cardiomyopathy and gut microbiota because of the vast similarities between pigs and humans. These similarities include, but are not limited to, the physiology and protein distribution of the skin, degradation of cardiac muscles, and gastrointestinal development. Researchers have used pigs for preclinical testing of invasive neurosurgical therapies such as deep brain stimulation (Gorny et al., 2013; Paek et al., 2015). The large size of the pig brain allows for the same instruments to be used for those in humans without the need for scaling (Orlowski et al., 2017). This preclinical work allows for the refinement of methods and safety measures to be investigated before implementation in humans.

An extensive atlas, similar to ones developed for the human and rodent brain, has been put together for identification of porcine brain structures (Félix et al., 1999). The porcine brain is considered a gyrencephalic neocortex that closely resembles that of a human (Villadsen et al., 2018). Using computer simulation software, the gyration of the brain results from gray matter developing at a faster rate than white matter (Tallinen et al., 2014). This finding has been confirmed using longitudinal MRI scans, with similarities between humans and pigs during the first months of brain development (Knickmeyer et al., 2008; Winter et al., 2011; Conrad et al., 2012). The folding of the brain allows for the increased complexity of neuronal networks and produces similarities in subcortical nuclei between pigs, nonhuman primates, and humans (Hofman, 1989; Larsen et al., 2004). Indeed, Larsen et al. (2004) found similarities in subthalamic nuclei shape and location between porcine brains, nonhuman primates, and humans. The similarities in structures between pigs, nonhuman primates, and humans could be a result of the rapid gyration of the brain pushing down on the subcortical regions to form distinct nuclei not seen in rodents.

The majority of brain growth, composition, and myelination occurs around birth in pigs, similar to human brain development (Dickerson and Dobbing, 1967; Conrad et al., 2012) and the white and gray matter densities in the porcine brain are similar to those in humans (Cullen et al., 2016). Sex-specific development also shares similarities between pigs and humans. In humans, females experience hippocampal development earlier than males but have a smaller maximum volume (Giedd et al., 1996). Conrad et al. (2012) found that female pig hippocampal development occurs 5 weeks before males. Moreover, the growth window for female hippocampal development is shorter than males. Typically, pigs live for 12–15 years of age, allowing for longitudinal studies to be conducted on the natural pathological development of neurodegenerative diseases.

The size of the pig allows for the use of modern preclinical and clinical imaging techniques used for humans without the need for scaling instrument size (Fang et al., 2005; Jørgensen et al., 2016; Villadsen et al., 2018). While humans do not necessarily need to be anesthetized for scans, pigs must be put under for accurate readings (Holm et al., 2016). However, protocols can be adjusted to accommodate this need as common drugs used for human anesthetics, such isoflurane and propofol, can be safely used on pigs (Holm et al., 2016). Positron emission topography (PET) scans have been used extensively in preclinical work on pigs for the development and testing of radioligand effectiveness prior to human use (Jørgensen et al., 2018; for review see Lind et al., 2007). These PET scans have revealed a number of similarities between human and pig monoaminergic systems. Within the serotoninergic system, pigs express numerous forms of 5-HT receptors within the caudate nucleus (5HT4, 5HT6, 5HT1D, and 5HT2C) and share a highly homologous 5HT1B to humans (Lind et al., 2007). Cumming et al. (2007) found that pigs and humans share similar serotonin neuron numbers within the raphe nucleus which differ greatly from rats (95,000 and 140,000 to 16,000, respectfully). Similarities in the dopamine systems have been observed with similar numbers of dendritic projections from the substantia nigra para compacta to the substantia nigra pars reticula seen in pigs, primates, and humans (Lind et al., 2007). Along with dendritic projections, D1/D2 receptor binding organization has been observed in pig brains, which resembles that seen in human brains (Minuzzi et al., 2006). Using FDOPA, an exogenous substrate to analyze DOPA decarboxylase levels during PET scans, it has been shown that pigs and humans share similarities in the regulation and metabolism of dopamine within the brain (Danielsen et al., 1999). Based on the reliability of using pigs in PET scans, further improvement and refinement of radio-labeled ligands used to detect specific pathological markers can take place without the necessary need for scaling from a rodent to a human.

## Use of Pigs to Study Alzheimer’s Disease

The first transgenic AD pig model was developed in 2009 by Kragh and colleagues. By using a human PDGFβ promoter fragment, commonly used in transgenic mice to induce human APP expression, they were able to detect APP6_95_*sw* transgene as early as 3 months in minipigs (Kragh et al., 2009). APP6_95_*sw* transgene mutation has been shown to lead to aberrant β-secretase cleavage of APP and increase production of Aβ40 and Aβ42 in mouse models (Holm et al., 2016; Jakobsen et al., 2016). Lee et al. (2017b) demonstrated a twofold increase in Aβ levels in the transgenic pig cortex through viral infection of human APP. Double transgenic minipigs have also been bred with APP6_95_*sw* and PSEN1M146I mutations and have shown to increase intraneuronal accumulation of Aβ42 (Jakobsen et al., 2016). These studies have established the genetic alteration required to express AD in pigs; however, the behavioral phenotype has yet to be established (Dolezalova et al., 2014). The establishment of the behavior that accompanies the pathology is a key component in translating what occurs in the pig model to the human form of the disease.

The hyperphosphorylation of tau and accumulation of NFT is considered a hallmark of AD. Rodents do not express the isoforms of tau known to lead to NFT development and so require the implantation of multiple transgenes or mutant human proteins (Oddo et al., 2003). Pigs, however, share various isoforms of tau found in the human brain. Of importance, pigs and humans contain two isoforms lacking exon 10, which has been shown to be less efficient at maintaining microtubule stability (Janke et al., 1999). The hyperphosphorylation of these isoforms of tau could lead to destabilization of the microtubules and the development of neurofibrillary tangles (Maccioni and Cambiazo, 1995). Further *in vivo* research is needed to investigate the development of neurofibrillary tangles from the increased levels of these tau isoforms.

## Use of Pigs Studying Traumatic Brain Injury

The similarities between the pig brain and human brain provide a unique opportunity to study the pathological effects of TBI. The development of the HYGE device allows for the analysis of neuropathological changes from rotational acceleration in an experimental setting initially used on nonhuman primates (Abel et al., 1978). With the ethical issues surrounding testing on nonhuman primates, there was a shift toward using the device on large animals such as pigs (Cullen et al., 2016). The HYGE device allows for the pathological profile of head trauma while controlling the biomechanical forces similar to what is observed in human TBI (Cullen et al., 2016; Vink, 2018). Compared to other head trauma models adopted from rodent studies, such as controlled cortical impact, fluid-percussion and weight-drop (reviewed in Xiong et al., 2013), the HYGE rotational model is considered to be the most accurate method to model human head trauma (Cullen et al., 2016).

Using the HYGE device, Smith et al. (1999a) were able to demonstrate axonal swelling in a pig model of TBI 1 day post-injury. This axonal swelling was marked by increased gliosis within the sulci of the injured brains. It is possible that the gliosis observed was due to the presence of astrocyte reactivity to damaged tissue (Levitt and Rakic, 1980); however, Smith and colleagues did not measure these levels. Measuring the levels of astrocytes following an impact using the HYGE device is crucial as increased astrocytic levels in the sulci has been observed following TBI and is considered to be a hallmark for the development of CTE (McKee et al., 2013).

Rotational acceleration of the porcine head results in accumulation of Aβ and hyperphosphorylated tau within the subcortical white matter (Smith et al., 1999a; Eucker et al., 2011; Wolf et al., 2017). Electrophysiological recordings from the CA1 field of the hippocampus revealed a significantly greater output in the injured brains, suggesting a possible compensatory mechanism due to decreased signal input from disrupted circuitry (Wolf et al., 2017). Interestingly, Wolf et al. (2017) found that while neuronal circuitry was altered, there was no accumulation of either Aβ or hyperphosphorylated tau within the hippocampus itself. Hyperphosphorylation of tau disrupts axonal transport and facilitates reactive oxygen species production, eventually leading to cell death (Stamer et al., 2002). This may indicate a slower time course of cellular degeneration as this subcortical structure only receives secondary-like biomechanical strain rather that focal impact strain (Sarntinoranont et al., 2012).

Johnson et al. (2016) performed rotational TBI on six-month old female Hanford pigs and found APP accumulation as early as 6 h post injury. Following injury, levels of neurofibrillary subtypes increased and were seen to co-label with APP. These increased levels of neurofibrillary subtypes showed a delayed response following injury, with levels reaching peak amounts between 48 and 72 h post injury. Interestingly, Ibrahim et al. (2010) also found APP accumulation within 6 h of rotational TBI; however, they did not find any changes in neurofilament levels, specifically NF68. Johnson et al. (2016) did find that in some axons, only one pathological phenotype was found (i.e., only NF-st, or APP, or the SNTF), so this could explain why there was no changes in the particular neurofilament found by Ibrahim et al. (2010). Future studies could investigate the difference in different phenotype expression and could lead to the development of effective clinical biomarkers. A follow up study conducted in 2018 found that along with APP accumulation in axons 48 h post injury, there was evidence of blood-brain barrier (BBB) permeability within 6 h of injury (Johnson et al., 2018). The axonal pathology within the white matter was seen to overlap with the areas with compromised BBB permeability.

Using a fluid percussion injury model in pigs, it was found that after a severe TBI there is evidence of diffuse axonal injury as evident from Aβ-positive staining within the white matter (Fritz et al., 2005). Along with diffuse axonal damage, evidence of necrosis was seen in this model. Shrunken neurons with darkened nuclei were observed in both the ipsilateral and contralateral hemispheres 24 h after impact (Fritz et al., 2005). Manley et al. (2006) observed alterations to microtubule integrity of neurons within the cortex 10 h after impact. This was achieved by using a controlled cortical impact model of TBI. The alteration of microtubule integrity was demonstrated by decreases in microtubule associated protein 2 (MAP2) levels and fragments of damaged neuronal structures. Along with damage to microtubules, there was evidence of degenerating neurons observed through increased Fluoro-Jade B-positive neurons within the impacted cortex (Manley et al., 2006). Table 1 describes the pathological findings using pig models of TBI previously discussed.

**Table 1:** Summary of TBI models in pigs and the pathology after impact.

Model	Time after injury	Pathology present	Reference
Rotational acceleration	1 day	Increased gliosis in sulci accompanied by axonal swelling.	Smith et al., 1999a
Fluid percussion injury	24 h	Subarachnoid hemorrhage around area of impact. Aβ-positive neurons and axonal damage in white matter. Shrunken neurons and evidence of necrosis.	Fritz et al., 2005
Controlled cortical impact	10 h	Perivascular hemorrhage around injury site. Damage to dendrites and decreased MAP2 staining. Fluro-Jade B-positive neurons mainly present in cell bodies, dendrites, and axons.	Manley et al., 2006
Rotational acceleration	6 h	Increased subarachnoid bleeding in moderate accelerated pigs. APP accumulation and axonal injury in diencephalon region. No changes in NF68.	Ibrahim et al., 2010
Rotational accelerations	6 h	Accumulation of Aβ and hyperphosphorylated tau in subcortical white matter.	Eucker et al., 2011
Rotational acceleration	6 and 48–72 h	Initial APP accumulation post-injury in the periventricular white matter. Development of neurofibrillary subtypes in subcortical white matter.	Johnson et al., 2016
Rotational acceleration	7 days	Electrophysiological recordings from CA1 field showed greater output. No accumulation of Aβ or hyperphosphorylated tau.	Wolf et al., 2017
Rotational acceleration	6 and 48 h	Blood brain barrier leakage after concussion. Axonal swelling and beading. BBB leakage overlapped with APP-positive-labeled axons in white matter.	Johnson et al., 2018

## Limitations

While the similarities between porcine and human brains suggest the use of pig brains in the study of neurodegenerative disorders, there remain limitations that need to be worked out. Rodents are relatively inexpensive, easy to care for in terms of food and housing, and a vast wealth of research has been collected devoted to understanding all aspects of their lifespan (Finnie, 2012; Eaton and Wishart, 2017). Pigs, on the other hand, require larger spaces for both experimentation and housing, require more resources for feeding, and there is a need for specialized knowledge of the animal to ensure proper health and lifestyle (Vink, 2018). Researchers have bred laboratory-purpose pigs such the Göttingen minipig in Germany, the Diannan Small-Ear minipig in China (Lind et al., 2007), and the previously mentioned APP transgenic pig (Kragh et al., 2009). These pigs, however, come at a cost for their controlled breeding. To get around this issue of cost, agricultural or “food-grade” pigs are commonly used as they are usually low cost and readily available (Lind et al., 2007). While this may seem like a better option, agricultural pigs produce a wide range of poorly defined breeds as a result of crossbreeding (Lind et al., 2007).

Another limitation in the use of pigs is the lack of sophisticated behavioral information within the literature. Pigs have a dynamic social system that follows a hierarchy of dominance within groups (Meese and Ewbank, 1973). It can be argued that pigs have social cognition as evident from their ability to recognize other pigs within a family group and displays of aggression toward outside members (Mendl et al., 2010). However, behavioral testing reveals high inter-individual variability during tests of memory integrity such as the object recognition task (Søndergaard et al., 2012). While this high inter-individual variability is something that is seen in human behavioral testing (Molleman et al., 2014), it presents a challenge when trying to categorize behaviors. Common rodent behavioral tests, such as the elevated plus maze, light/dark box, and classical conditioning procedures have been used with pigs (for review, see Murphy et al., 2014). Initial work on these behaviors has revealed a contradiction to what is seen in rodent studies; pigs tend to spend equal time in both light and dark conditions of the light/dark box and elevated plus maze showing a lack of anxiety or fear (Andersen et al., 2000). However, pigs have been shown to learn Go/NoGo tasks successfully (Scollo et al., 2014) with evidence to suggest the use of striatal dopamine system for object-directed exploratory behavior (Lind et al., 2005). Taken together, the use of pigs for studying general behavioral research still requires additional research and understanding before using pigs for the study of neurodegenerative diseases.

## Conclusion

While the porcine brain presents some unique features to study neuropathology, further work is needed to increase the validity of using pigs in the study of neurodegenerative diseases. The gyrencephalic nature of the brain allows for the investigation of the pathological outcomes that may parallel those seen in humans. The longer life of the pig and the effectiveness of using PET scans provide a unique opportunity to better understand development of biomarkers to detect early changes in AD and the resulting changes following TBI (Kondo et al., 2015; Eaton and Wishart, 2017). The longer life-span of a pig allows for longitudinal studies to assess the natural development of AD and long-term consequences of TBI, at the increased expense of housing and caring for these animals. While the inter-species behavioral variability may make it difficult to assess the complex behavioral abnormalities associated with neurodegenerative diseases (Eaton and Wishart, 2017), this variability certainly follows the human condition more closely. Methodological and behavioral measures used from well-accepted rodent models can be adapted and modified to help establish the use of pigs as a potential translational model for greater understanding on these diseases and the better development of therapeutics.

## Author Contributions

Contribution of BH included the research and writing of this review paper. Contribution of MH included the generous revisions of the final edition of the review paper.

### Conflict of Interest Statement

The authors declare that the research was conducted in the absence of any commercial or financial relationships that could be construed as a potential conflict of interest.
